# Prospective study of TACE combined with sorafenib *vs* TACE combined with ^125^I seed implantation in the treatment of hepatocellular carcinoma with portal vein tumor thrombus and arterioportal fistulas

**DOI:** 10.3389/fonc.2022.977462

**Published:** 2022-10-05

**Authors:** Xiao-Hui Zhao, Hang Yuan, Wei-Li Xia, Li-Lin Zhang, Zhen Li, Guang-Shao Cao, Hai-Liang Li, Wei-Jun Fan, Hong-Le Li, Chen-Yang Guo, Quan-Jun Yao, Wen-Bo Zhu, Hong-Tao Hu

**Affiliations:** ^1^ Department of Minimal-Invasive Intervention, The Affiliated Cancer Hospital of Zhengzhou University & Henan Cancer Hospital, Zhengzhou, China; ^2^ Yangtze University Health Science Center, Jingzhou, China; ^3^ Department of Interventional Radiology, The First Affiliated Hospital of Zhengzhou University, Zhengzhou, China; ^4^ Department of Intervention, Henan Provincial People's Hospital, People's Hospital of Zhengzhou University, Zhengzhou, China; ^5^ Imaging and Interventional Department, Sun Yat-sen University Cancer Center, Guangzhou, China; ^6^ The Affiliated Cancer Hospital of Zhengzhou University & Henan Cancer Hospital, Zhengzhou, China

**Keywords:** hepatocellular carcinoma, arterioportal fistulas, portal vein tumor thrombus, ^125^ I seed, transarterial chemoembolization, sorafenib

## Abstract

**Purpose:**

To compare the efficacy of TACE combined with sorafenib and TACE combined with ^125^I seed implantation in the treatment of hepatocellular carcinoma (HCC) with portal vein tumor thrombus (PVTT) combined with arterioportal fistulas (APFs), and discuss the efficacy and safety of TACE combined with ^125^I seed implantation.

**Patients and methods:**

Between January 2017 and December 2018, the clinical data of patients with HCC complicated with PVTT and APFs who were admitted to the Affiliated Cancer Hospital of Zhengzhou University, First Affiliated Hospital of Zhengzhou University, and Henan Provincial People’s Hospital were prospectively collected. The patients were divided into the TACE+sorafenib (TACE-S) group based on their treatment willingness. There were 26 and 32 patients in the TACE-S and TACE-^125^I groups, respectively. Both groups of patients underwent APFs occlusion during TACE therapy. The embolization effect of APFs was observed and recorded in the two groups, the efficacy of intrahepatic lesions and PVTT was evaluated, and the effects of different treatment methods on the efficacy were analysed.

**Results:**

All patients completed the 3 months follow-up. The improvement rates of APFs in TACE-S and TACE-^125^I groups were 30.77% (8/26) and 68.75% (22/32), respectively, and difference was statistically significant (χ2 = 8.287, P=0.004). The median survival time of TACE-S and TACE-^125^I groups was 8.00 months and 12.8 months, respectively (χ2 = 7.106, P=0.008). Multivariate analysis showed that the PVTT subtype (IIa/IIb) and treatment method (TACE-S or TACE-^125^I) were independent factors affecting the recanalization of APFs in patients (P<0.05).

**Conclusion:**

For patients with HCC with PVTT and APFs, TACE combined with ^125^I seed implantation can effectively treat portal vein tumor thrombus, thereby reducing the recanalization of APFs and prolonging the survival time of patients.

## 1 Introduction

The proportion of portal vein tumor thrombus (PVTT) in patients with advanced hepatocellular carcinoma (HCC) in China is high, ranged 44%–62.2% (1, 2). PVTT has been recognized as an independent risk factor for poor prognosis in HCC patients (3). Median overall survival (OS) was only 2.7-4 months in patients with HCC with PVTT with best supportive care only, compared with 10-24 months in patients without PVTT (4). Arterioportal shunts have been reported in 27-63.2% (5) of advanced HCC cases and may be caused by PVTT. The emergence of arterioportal fistulas (APFs) increases the risk of serious complications such as esophageal varices, ascites, and hepatic encephalopathy (6, 7), which seriously affects the prognosis and survival of patients.

The Barcelona Clinic Liver Cancer (BCLC) system recommends sorafenib alone for HCC patients with PVTT (8). In the Asia-Pacific region, transarterial chemoembolization combined with sorafenib (TACE-S) is the more commonly used treatment for such patients (9, 10). In TACE procedures, APFs are frequently seen by digital subtraction angiography (DSA) (7), which is also the gold standard for diagnosis. In addition, multi-slice CT angiography (MSCTA) can also detect the presence of APFs (11, 12). However, because of poor control of PVTT, even if the APFs are blocked, most patients recanalize the APFs on subsequent follow-up examinations.

In addition, direct puncture implantation of ^125^I seeds can be used to treat tumor thrombus in portal vein branches (12–14). A prospective study in China reported that TACE-^125^I was superior to TACE-S in the treatment of HCC patients with branch portal vein tumor thrombus (12). There are no studies to prove that implantation of ^125^I in PVTT can lead to favorable APFs response. Therefore, we designed this prospective, non-randomized controlled study to compare the efficacy and safety of TACE combined with sorafenib and TACE combined with PVTT ^125^I seed implantation for the treatment of HCC patients with PVTT and APFs.

## 2 Material and methods

### 2.1 Patient information

This prospective controlled study complies with the ethical guidelines of the World Medical Association Declaration of Helsinki. The overall clinical trial has been registered in the Chinese Clinical Trials Database (number ChiCTR-ONN-16007929). This study was reported as a subgroup of the overall clinical trial from which the data were derived. All patients signed informed consent and had the right to withdraw from the study at any time.

**Figure 1:**
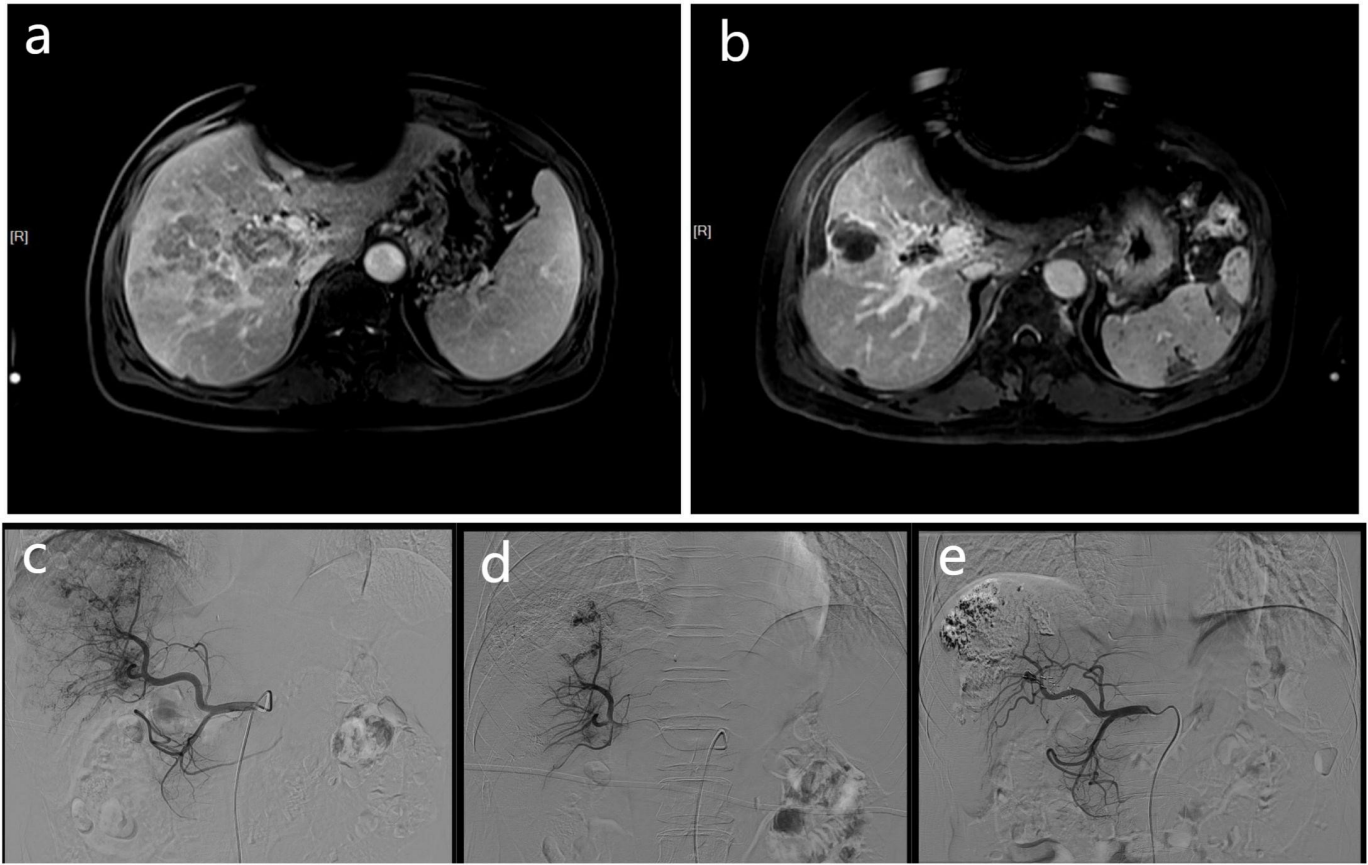
These MR and DSA images show the imaging data of a 68-year-old men with HCC complicated by portal vein tumor thrombosis in the right branch. Contrast-enhanced MR scan showing a tumor thrombus in right portal vein **(A)**. The contrast-enhanced MR of the patient 3 months later shows a significant reduction in PVTT volume **(B)**, and the previously blocked portal vein due to PVTT also restores blood flow. At the same time, according to the mRECIST criteria, there was no activity in the intrahepatic lesions, and no activity was found in the PVTT, which was judged as CR. The DSA image of the patient undergoing embolization of the APFs and obvious portal vein development can be seen during angiography of the proper hepatic artery **(C)**. The angiographic image of the patient taken at a time after treatment, showing that the APFs are completed by sealing **(D)**. A follow-up DSA image of the patient three months later showed no APFs **(E)**.

Between January 2017 and December 2018, 127 patients with HCC and PVTT were treated in our department. A total of 58 patients were found to have APFs during hepatic angiography, and they were immediately included in this study. The inclusion criteria were as follows (1): Diagnosis according to the criteria of the European Association for the Study of Liver Disease/American Association for the Study of Liver Disease (15), diagnosis of HCC complicated with PVTT (**Figure 1**), and only included in Cheng’s classification Type II PVTT (16); (2) Hepatic artery DSA confirmed APFs; (3) Child-Pugh class A or B. Exclusion criteria: (1) patients who had received anti-tumor therapy such as surgery, radiofrequency or microwave ablation, systemic chemotherapy, and intra-arterial chemoinfusion, or TACE; (2) severe concomitant diseases, such as severe heart failure or respiratory diseases; (3) hepatic encephalopathy or extrahepatic metastasis; (4) Child-Pugh grade C; and (5) uncorrectable renal and coagulation dysfunction.

Based on fully introducing the two treatment methods and respecting the wishes of the patients, they were divided into two groups. A group of patients received TACE + sorafenib (TACE-S) treatment, which consisted of a total of 26 cases. The other group received TACE+PVTT ^125^I seed implantation (TACE-^125^I), which consisted of a total of 32 patients. A total of 58 patients were included in the study.

### 2.2 TACE procedure

As previously reported (12, 14), routine celiac arteriography and hepatic arteriography were performed to confirm the diagnosis of HCC and the specific conditions of the APFs. If the presence of APFs was confirmed, a 2.7 F coaxial microcatheter (Terumo, Japan) was used to select the corresponding artery for superselective arteriography to determine the location of the APFs and determine the flow. After hepatic arteriography, there are two types of embolization according to whether the microcatheter can pass through the APFs area: (1) if the microcatheter can pass through the APFs area, tumor embolization should be performed first. A volume of 5–20 ml Lipiodol (Lipiodol Ultrafluide, Laboratoire Guerbet, France) and doxorubicin (50–75 mg/m2) (Haizheng Pharmaceutical Co. Ltd., China) were mixed to prepare a lipiodol emulsion. After tumor chemoembolization was completed, the microcatheter was returned to the APFs area, and polyvinyl alcohol of different diameters (polyvinyl alcohol foam embolization particles; Cook Medical Inc., Bloomington, IN, USA) was selected to embolize the fistula; (2) if the microcatheter could not pass through the APFs area, chemoembolization was performed in a certain area of the supplying artery, and the target tumor and APFs were simultaneously embolized. When it was found that APFs still existed during the re-imaging, a spiral steel ring (China, Cook Medical Trading Co., Ltd.) was chosen. Angiography showed that the APFs disappeared or most of the shunt disappeared, and the operation was ended.

### 2.3 Sorafenib treatment

All patients in the TACE-S group started sorafenib (400 mg, bid) 3-7 days before the first TACE treatment. Therefore, patients should continue sorafenib treatment. If there is an obvious clinical toxicity related to the treatment, the dose can be reduced or discontinued depending om the situation. After the toxicity subsided or disappeared, the original dose or resumed medication was restored.

### 2.4 ^125^I seed implantation procedure

The liver functions of patients in the TACE-^125^I group were followed up 3-7 days after the first TACE treatment. An enhanced CT scan was performed before surgery, the image was imported into the treatment planning system (TPS) (FTT Technology Ltd. Co, Beijing, China), the ^125^I seed implantation plan was planned to calculate the formula dosage, number, spatial distribution, intensity of radioactivity, and matched peripheral dosage of seeds before implantation so that 95% of the tumor target volume reached the prescribed dose, and the target volume ratio reaches 1.5–2.0. According to the TPS system, an 18 G seed implantation needle was used to puncture the target lesion, and ^125^I seeds were implanted in different layers and positions of the tumor. According to the spacing of the 5 mm cloth source, the particle distribution was uniform. A CT scan was performed immediately after surgery to observe the particle distribution.

### 2.5 Evaluation indicators

According to the time of appearance of AFPs, they are divided into three categories: (1) Mild: no fistula shape is shown on angiography, and when bolus injection of lipiodol is used for embolization, the small branches of the portal vein can be seen. (2) Moderate: the main or branch of the portal vein is visualized in the middle and late stages of tumor staining. (3) Severe: the portal vein is visible when the main and branches of the hepatic artery are visualized. At this time, tumor staining was absent or was at an early stage.

MSCTA diagnostic criteria for AFPs (11, 12): (1) the main portal vein or first-order branches in the hepatic arterial phase are visualized early, while the splenic vein and superior mesenteric vein have not been enhanced; (2) the hepatic arterial phase peripheral portal vein secondary or secondary and distal branches are visualized early, while the proximal main portal vein and left and right branches are not yet enhanced. The diagnostic criteria for APFs include the early development of hepatic vein branches in the hepatic arterial phase, while the portal vein and liver parenchyma have not yet been enhanced.

Criteria for evaluating the therapeutic effect of APFs were as follows: (1) changes in APFs classification: ① Cure, APFs disappear completely; ② Relief, APFs degree is reduced or time delay occurs; ③ Stabilize, APFs level remains unchanged; ④ Progress, APFs level increase in flow rate. DSA angiography images of all patients were re-examined 3 months after the first treatment, and the effect of fistula embolization was observed according to the arterial angiography images. Among them, ① and ② were considered effective, and ③ and ④ were considered ineffective. (2) Changes in liver function indices, including Child-Pugh grade and total bilirubin and albumin levels, were found.

The modified response evaluation criteria in solid tumors (mRECIST) were used to evaluate the efficacy of tumor embolization three months after the first treatment (17), including complete response (CR), partial response (PR), stable disease (SD), and progressive disease (PD). Intrahepatic tumor lesions and portal vein tumor thrombus were evaluated separately.

### 2.6 Study objectives and follow-up

All patients were re-examined 4-6 weeks after the first TACE with plain upper abdominal MRI plus dynamic enhancement. Repeat TACE is feasible for residual tumor lesions. APF assessment of APFs is based on DSA angiography as the gold standard. Some patients no longer receive TACE after their condition has stabilized. Therefore, there is no direct evidence to prove recanalization of APFs, and the judgment is based on the performance of the MSCTA.

The improvement rates of APFs was defined as the percentage of responders (① and ②). The disease control rate (DCR) was defined as the percentage of CR+PR+SD patients. The time from treatment to the last follow-up or death was defined as overall survival (OS). The main objectives were to evaluate the improvement rates of APFs, changes in liver function indices, and DCR of intrahepatic lesions and PVTT after the first TACE. The secondary endpoint was OS.

### 2.7 Statistical analysis

All statistical analyses were performed using SPSS version 25.0 (SPSS, Chicago, IL, USA). To determine significant differences between groups, Student’s t-test, chi-squared test, or Fisher’s exact test was used. Factors affecting recanalization of APFs were analysed using univariate and multivariate logistic regression analyses. Survival analysis was performed using the Kaplan-Meier method, and an OS curve was drawn. The difference in survival between the two groups was analysed using the log-rank test. Statistical significance was set at P< 0.05.

## 3 Results

### 3.1 Basic characteristics

A comparative analysis of the TACE-S and TACE-^125^I groups is shown in (**Table 1**). There were no significant differences in gender, age, ECOG score, Child-Pugh score, tumor number, largest tumor diameter, total tumor diameter, type of PVTT (IIa or IIb), AFP level, or blood test results (P> 0.05).

**Table 1:** Comparison of general data of patients in the TACE-^125^I group and the TACE-S group.

Variable	Grading	TACE-^125^I groupn=32	TACE-S groupn=26	P value
Gender	Male	27 (84.4)	23 (88.5)	0.947
	Female	5 (15.6)	3 (11.5)	
Age	≤60	17 (53.1)	13 (50.0)	1.000
	>60	15 (46.9)	13 (50.0)	
ECOG Score	0	9 (28.1)	12 (46.2)	0.252
	1	23 (71.9)	14 (53.8)	
Child-Pugh classification	Class A	29 (90.6)	20 (76.9)	0.285
	Class B	3 (9.4)	6 (23.1)	
Number of liver tumors	1	10 (31.2)	12 (46.2)	0.373
	≥2	22 (68.8)	14 (53.8)	
Maximum tumor diameter	<60	19 (59.4)	18 (69.2)	0.616
	≥100	13 (40.6)	8 (30.8)	
Total tumor diameter	<100	24 (75.0)	23 (88.5)	0.335
	≥100	8 (25.0)	3 (11.5)	
Tumor location	Single leaf	8 (25.0)	9 (34.6)	0.610
	Futaba	24 (75.0)	17 (65.4)	
PVTT type	IIa	18 (56.2)	17 (65.4)	0.662
	IIb	14 (43.8)	9 (34.6)	
APFs classification	Mild	17 (53.1)	13 (50.0)	0.949
	Moderate	11 (34.4)	10 (38.5)	
	Severe	4 (12.5)	3 (11.5)	
AFP(ng/ml)	<400	15 (46.9)	16 (61.5)	0.396
	≥400	17 (53.1)	10 (38.5)	
TBL (g/L)		21.51 (11.13)	19.24 (11.71)	0.453
ALB (μmol/L)		38.77 (5.31)	37.96 (4.54)	0.540
PT(s)		12.97 (1.17)	13.30 (1.80)	0.415
WBC(10×12/L)		4.94 (1.87)	5.31 (1.60)	0.423
RBC(10×9/L)		4.33 (0.50)	4.17 (0.49)	0.238
HGB(g/L)		132.53 (15.83)	130.12 (12.38)	0.528

Unless otherwise indicated, data are presented as numbers of patients.

TACE, transarterial chemoembolisation; ECOG, Eastern Cooperative Oncology Group; PVTT, portal vein tumor thrombosis; AFP, alpha-fetoprotein; TBL, total bilirubin; ALB, albumin; PT, prothrombin time; WBC, white blood cell; RBC, red blood cell; HGB, haemoglobin.

### 3.2 Evaluation of the therapeutic effect of APFs

All 58 patients were followed up for 3 months after treatment, and there were statistically significant differences in APFs grading between the two groups before treatment and 3 months after treatment (**Table 2**). After 3 months of treatment, 22 patients in the TACE-^125^I group were effective for APFs occlusion, while 10 were ineffective. In the TACE-S group, 8 were effective and 18 were ineffective. The improvement rates of APFs in the TACE-S group and TACE-^125^I group were 30.77% (8/26) and 68.75% (22/32), respectively, and the difference was statistically significant (χ2 = 8.287, P=0.004) (**Table 2**). There were no differences in Child-Pugh grade and albumin and bilirubin levels between the TACE-S and TACE-^125^I groups before and after treatment (**Table 3**).

**Table 2:** Comparison of APFs grades before and 3 months after treatment between the TACE-^125^I group and the TACE-S group.

Groups	Before treatment				After treatment				χ ^2^	P *value*
	None	Mild	Moderate	Severe	None	Mild	Moderate	Severe		
TACE-^125^I group (n=32)	0	17	11	4	16	11	1	4	29.010	0.001
TACE-S group (n=26)	0	13	10	3	5	5	12	4	8.750	0.029
X^2^	0.158				17.317					
P value	0.949				0.001					

**Table 3:** Comparison of Child-Pugh classification, as well as total bilirubin and albumin levels, before treatment and 3 months after treatment in the TACE-^125^I group and the TACE-S group.

Variable		TACE-S group			TACE-^125^I group		
		Before therapy	After treatment	P value	Before therapy	After treatment	P value
Child-Pugh classification	A	20 (76.9)	18 (69.23)	0.532	29 (90.6)	30 (93.75)	1.000
	B	6 (23.1)	8 (30.77)		3 (9.4)	2 (6.25)	
Total bilirubin (g/L)		19.24 ± 11.71	18.37 ± 10.12	0.766	21.51 ± 11.13	18.7 7 ± 10.17	0.291
Albumin (μmol/L)		37.96 ± 4.54	37.72 ± 5.16	0.866	38.77 ± 5.31	38.62 ± 4.81	0.907

### 3.3 Efficacy assessment of intrahepatic lesions and PVTT

The DCR of intrahepatic lesions in the TACE-^125^I group and TACE-S group were 84.38% and 60.71%, respectively, and this difference was statistically significant (χ2 = 4.275, P=0.039) (**Table 4**). The DCR of PVTT in TACE-^125^I group and TACE-S group were 81.25% and 53.57% , respectively, and the difference was statistically significant (χ2 = 5.287, P= 0.021) (**Table 5**).

**Table 4:** Efficacy assessment for intrahepatic lesions.

	TACE-^125^I group	TACE-S group	χ ^2^	P *value*
CR	1 (3.12%)	0 (0.0%)		
PR	12 (37.50%)	7 (25.00%)		
SD	14 (43.75%)	10 (35.71%)		
PD	5 (15.63%)	11 (39.29%)		
DCR	84.38%	60.71%	4.275	0.039

**Table 5:** Efficacy assessment for PVTT.

	TACE-^125^I group	TACE-S group	χ ^2^	P *value*
CR	1 (3.13%)	0 (0.0%)		
PR	13 (40.635%)	6 (21.43%)		
SD	12 (37.50%)	9 (32.14%)		
PD	6 (18.75%)	13 (46.43%)		
DCR	81.25%	53.57%	5.287	0.021

Unless otherwise indicated, data are numbers of patients.

CR, complete response; DCR, disease control rate; PD, progressive disease; PR, partial response; PVTT, portal vein tumor thrombosis; SD, stable disease; TACE, transarterial chemoembolisation.

### 3.4 Analysis of factors affecting APFs recanalization

Univariate and multivariate analyses showed that PVTT subtype (IIa/IIb) and treatment method (TACE-S or TACE-^125^I) were factors affecting the recanalization of APFs (**Table 6**).

**Table 6:** Multivariate Analysis of Influencing APFs Recanalization.

variable	RR	95% confidence interval	P value
PVTT type (IIa/IIb)	15.88	2.99-84.34	0.001
Treatment (TACE-S group/TACE-^125^I group)	13.07	2.54-67.16	0.002

The binary logistic regression model was used.

### 3.5 Subsistence analysis

**Figure 2:**
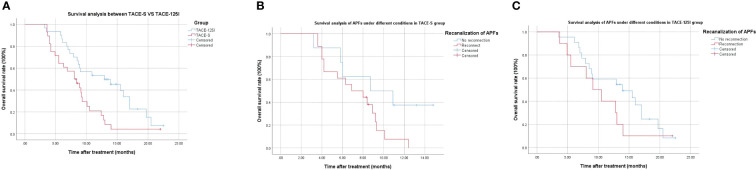
Survival curve of patients in TACE-S group and TACE-^125^I group.

As of 31 December 2020, the 12-month survival rates were 12.2% in the TACE-S group and 53.1% in the TACE-^125^I groups. The median overall survival (mOS) of the TACE-S group and TACE-^125^I group were 8.00 months and 12.8 months, respectively (χ2 = 7.106, P=0.008) (**Figure 2A**). Survival analysis of patients with successful APFs recanalization and occlusion in each group. TACE-S group: 8.7 months *vs* 6.9 months (χ2 = 3.155, P=0.08) (**Figure 2B**); TACE-^125^I group: 13.9 months *vs* 9.1 months (χ2 = 1.454, P=0.228) (**Figure 2C**).

### 3.6 Adverse reactions

Some patients experienced adverse reactions related to TACE treatment and post-embolization syndromes of varying degrees, such as abdominal pain, nausea, and vomiting. After to 3-7 days of symptomatic treatment, the patient’s symptoms were relieved. The overall incidence of adverse events or toxic effects related to sorafenib use was 84.61% (22/26). The most common grade 3 to 4 adverse events were diarrhoea (7.69%, 2/26) and hand-foot skin reactions (3.85%, 1/26). Adverse effects of PVTT^125^I seed implantation included haemorrhage and pneumothorax, which were relieved by symptomatic treatment. No serious adverse reactions, such as surgery-related deaths, were observed.

## 4 Discussion

Previous studies have shown that PVTT is an independent risk factor for recanalization (18). In our study, the APFs occlusion rate of patients in the TACE-^125^I group was high (68.75%) and one-time intraoperative occlusion rate was 100%. Our study analysed the occlusion effect of APFs 3 months after TACE, and confirmed that compared with TACE-S, TACE combined with portal vein tumor thrombus ^125^I seed implantation can control the recanalization of APFs.

Previous studies have shown that embolic materials for the treatment of hepatic arterial shunt (APS) include ethanol-soaked gelatin sponge (ESG) and polyvinyl alcohol (PVA) particles. On the basis of TACE-based therapy, aggressive and thorough APS embolization during surgery can reduce the occurrence of fistula recanalization (19, 20). In our study, all patients achieved complete occlusion by using PVA particles during TACE procedure. The final results showed that the recanalization rate of APFs in the TACE-S group was still high, even when the APFs were blocked with the same material. The results of the APS study combined with PVTT showed that there were differences in the survival of patients with different PVTTs (21). Therefore, for patients with APF and PVTT, it is important to control the progression of PVTT after complete occlusion therapy. This will further prolong patient survival.

In previous studies, APS improvement was shown to be an independent prognostic factor (6). However, most studies focus on materials for the treatment of APS, ignoring the specificity of APS with PVTT. As we believe, fistula and tumor thrombus are mutually causal and mutually reinforcing results. The mechanism may be: the formation of PVTT is mainly related to portal venous reflux. About 47%-63% of liver cancers are associated with hepatic arteriovenous fistula (22), mainly the hepatic artery-portal communicating branch. 90% of the blood supply of HCC comes from the hepatic artery, and the liver cancer cells are directly injected into the small branch of the portal vein with low pressure through the blood flow of the hepatic artery with higher pressure, and stay and implant in the portal vein to form PVTT. The formation of PVTT results in the obstruction of the portal vein, resulting in the opening of extensive anastomotic branches between the hepatic artery and the portal vein in normal liver tissue to form fistulas, further aggravating the occurrence of intrahepatic decompensation events.

In terms of related indicators before and after treatment, the results of this study showed that the proportion of patients with Child-Pugh A grade in the TACE-^125^I group did not change significantly (90.6% *vs* 93.75%) 3 months after treatment, and the proportion of those with Child-Pugh B grade in the TACE-S group increased (23.1% *vs*. 30.77%), although no statistical difference was observed. However, the control of PVTT by ^125^I particles relieves clogging of the portal vein, and is more important for the relief of liver function, which is consistent with previous studies (12).

Multivariate logistic analysis showed that the type of PVTT and treatment were prognostic factors affecting recanalization of APFs. In our study, there were statistically significant differences in the DCR for both intrahepatic lesions and PVTT. The median survival times of patients in the two groups were 8.00 months and 12.80 months, respectively. Compared with previous results, the tumor response in our study was poor, which may be related to the effect of APFs on TACE efficacy (12, 23). In addition, studies have shown that DEB-TACE and Y-90 radioembolization have good efficacy and safety in HCC patients with PVTT (24, 25). However, Y-90 radioembolization is not feasible for the appearance of APFs.

Further analysis of our study revealed the results. Under the same treatment modalities, the survival of patients with fistula recanalization was worse than that of patients with fistula closure (TACE-S group: 8.7 months *vs* 6.9 months, P=0.08; TACE-^125^I group: 13.9 months *vs* 9.1 months, P=0.228), although no statistical difference was shown (P>0.05). This may be related to our limited sample size. APFs interact with PVTT to make the prognosis of patients worse, and further research is needed to prove this inference.

In terms of safety, previous studies have also shown that TACE combined with sorafenib has no unexpected toxicities (23), and this study did not show any additional adverse reactions. The adverse reactions related to ^125^I seed implantation are mainly caused by puncture and subcapsular haemorrhage. Only part of the puncture route passes through the lung tissue, and the pneumothorax caused by it is mild.

This study has certain limitations. First, although this study is a prospective multicentre study, it is a non-randomized control, it may cause statistical bias. Additionally, the small sample size is an important limitation. Secondly, considering that previous studies have shown that different materials have different effects on APFs (19, 20), and to avoid confounding factors, all patients were treated with PVA particles. Further research on the efficacy of different blocking materials for APFs is necessary after actively controlling PVTT.

## 5 Conclusion

In conclusion, from the perspective of fistula recanalization, this study found that in patients with HCC complicated with PVTT and APFs, TACE combined with ^125^I seed implantation can more effectively reduce the occurrence of APFs recanalization. In such patients, the control of PVTT progression may be more important. TACE combined with portal vein tumor thrombus ^125^I seed implantation is effective and safe in the treatment of advanced HCC patients with type II PVTT, and can significantly prolong patient survival.

## Data availability statement

The raw data supporting the conclusions of this article will be made available by the authors, without undue reservation.

## Ethics statement

This study was reviewed and approved by The study protocol conformed to the ethical guidelines of the World Medical Association Declaration of Helsinki, was approved by the Ethics Committee of our institution (2016ct005) and was registered in the Chinese Clinical Trials Database (number ChiCTR-ONN-16007929). This study was reported as a subgroup of the overall clinical trial from which the data were derived. All patients provided written informed consent for participation in this study. The patients/participants provided their written informed consent to participate in this study. Written informed consent was obtained from the individual(s) for the publication of any potentially identifiable images or data included in this article.

## Author contributions

Conception and design: H-TH. Patient selection and treatment: X-HZ, HY, W-LX, H-TH,G-SC, ZL, Hai-LL, C-YG, Q-JY and W-BZ. Data collection, analysis, and interpretation: X-HZ, HY, W-LX and L-LZ. Data interpretation: X-HZ, H-TH, and Hai-LL. Undertook steering committee activities and critical statistical processing: H-TH and Hai-LL . Manuscript writing: X-HZ, HY, W-LX and L-LZ. Manuscript reviewing: H-TH, W-JF and Hong-LL . All authors contributed to the article and approved the submitted version.

## Funding

This study was funded by Guerbet France, Henan Province Natural Science Foundation (212300410403), Henan Province Medical Science and Technology Research Project (201701032) and National Science and Technology Major Project of the Ministry of Science and Technology of China (2018ZX10303502). Medical Education Research Project of Henan Province (Wjlx2021334).

## Conflict of interest

The authors declare that the research was conducted in the absence of any commercial or financial relationships that could be construed as a potential conflict of interest.

## Publisher’s note

All claims expressed in this article are solely those of the authors and do not necessarily represent those of their affiliated organizations, or those of the publisher, the editors and the reviewers. Any product that may be evaluated in this article, or claim that may be made by its manufacturer, is not guaranteed or endorsed by the publisher.
